# Infection and HLA-G Molecules in Nasal Polyposis

**DOI:** 10.1155/2014/407430

**Published:** 2014-03-06

**Authors:** Roberta Rizzo, Nicola Malagutti, Daria Bortolotti, Valentina Gentili, Antonella Rotola, Enrico Fainardi, Teresa Pezzolo, Claudia Aimoni, Stefano Pelucchi, Dario Di Luca, Antonio Pastore

**Affiliations:** ^1^Section of Microbiology and Medical Genetics, Department of Medical Sciences, University of Ferrara, Via Luigi Borsari, 46, 44121 Ferrara, Italy; ^2^Operative Unit of Otolaryngology, St. Anna Hospital, Via A. Moro, 844124 Ferrara, Italy; ^3^Operative Unit of Neuroradiology, St. Anna Hospital, Via A. Moro, 844124 Ferrara, Italy

## Abstract

Sinonasal polyposis (SNP) is a chronic inflammatory pathology with an unclear aetiopathogenesis. Human papillomavirus (HPV) infection is one candidate for the development of SNP for its epithelial cell trophism, hyperproliferative effect, and the induction of immune-modulatory molecules as HLA-G. We enrolled 10 patients with SNP without concomitant allergic diseases (SNP-WoAD), 10 patients with SNP and suffering from allergic diseases (SNP-WAD), and 10 control subjects who underwent rhinoplasty. We analyzed the presence of high- and low-risk HPV DNA and the expression of membrane HLA-G (mHLA-G) and IL-10 receptor (IL-10R) and of soluble HLA-G (sHLA-G) and IL-10 by polyp epithelial cells. The results showed the presence of HPV-11 in 50% of SNP-WoAD patients (OR:5.5), all characterized by a relapsing disease. HPV-11 infection was absent in nonrelapsing SNP-WoAD patients, in SNP-WAD patients and in controls, supporting the hypothesis that HPV-11 increases risk of relapsing disease. HPV-11 positive SNP-WoAD patients presented with mHLA-G and IL-10R on epithelial cells from nasal polyps and showed secretion of sHLA-G and IL-10 in culture supernatants. No HLA-G expression was observed in HPV negative polyps. These data highlight new aspects of polyposis aetiopathogenesis and suggest HPV-11 and HLA-G/IL-10 presence as prognostic markers in the follow-up of SNP-WoAD.

## 1. Introduction

Sinonasal polyposis (SNP) is a chronic inflammatory pathology characterized by the formation of nasal polyps at the level of the nasal cavity and paranasal sinuses, resulting from an edematous multifocal degeneration of the mucosa. These benign lesions affect approximately 1–4% of the general population, with a slight preference towards elderly men [1]. They are most often treated with steroids or surgery, although nasal polyps removed by surgery have a 70% chance of recurrence. The mechanisms for polyps development are not clear, even though allergies, asthma, aspirin-sensitive individuals, and chronic sinus infections are frequently associated [2, 3]. Viral infection has been postulated to be one important aetiological factor in the pathogenesis, progression, and recurrence of nasal polyps [4], with human papillomavirus (HPV) infection as a candidate for the development of nasal polyps [5, 6].

HPV is a small unenveloped double-stranded DNA virus with strict tissue and species specificity. Many different papillomaviruses infect animals, and over 150 genotypes have been so far identified in humans. Papillomaviruses infect squamous epithelia as skin and mucosae. The mucosal types of HPV fall in two groups: low-risk types (LR-HPV) (mainly HPV-6 and -11), which induce benign cell hyperproliferation, and the high-risk types (HR-HPV), which lead to malignancies as invasive cervical carcinoma, anal cancer, and oropharyngeal carcinomas.

Previous studies have shown that HPV infection may be associated with human nasal polyposis, such as inverted papilloma [5, 6]. However, the role of HPV infection and type in SNP has not been clearly demonstrated. Moreover, HPV infection is often transient and the host immune system could counteract viral invasion leading to lesion regression [7], as the host immune system is able to counteract the infection. On the other hand, HPV is able to downregulate host immune system [8], blocking interferon response, antigen processing, and presentation [9] and modifying human leukocyte antigen (HLA)-G expression [10, 11].

HLA-G is a nonclassical HLA class I molecule with a physiological tissue-restricted distribution in cytotrophoblast [12], amniotic cells [13], thymus [14], and endothelial cells of chorionic blood vessels [15]. HLA-G molecules are generated by an alternative splicing of the primary transcript of the gene; HLA-G exists as four-membrane bound (HLA-G1, -G2, -G3, and -G4) and three soluble isoforms (HLA-G5, -G6, and -G7) [16, 17]. HLA-G exhibits low allelic polymorphisms in comparison with classical HLA class I genes, with only 50 alleles (IMGT HLA database, December 2013) and 16 proteins. HLA-G is characterized by tolerogenic functions, inducing apoptosis of activated CD8+ T cells [18], promoting T regulatory cells [19], modulating the activity of natural killer cells [20] and of dendritic cells [21], and blocking allocytotoxic T lymphocyte response [22]. These immunoregulatory functions are mediated by the interaction of HLA-G molecules with specific inhibitory receptors: ILT-2 (LILRB1/CD85j), ILT-4 (LILRB2/CD85d), CD8, and KIR2DL4 (CD158d) expressed by immune cells [23]. We previously demonstrated a generalized defect in sHLA-G production by peripheral blood mononuclear cells of SNP patients [23] that seems to be mainly related to the interleukin (IL)-10/HLA-G pathway. IL-10 is one of the main HLA-G inducers [24] but it does not seem to be able to upmodulate sHLA-G production in SNP patients despite the elevated/normal production of IL-10. Since previous studies reported an involvement of HLA-G molecules in HPV-associated tumours [10, 11, 25–27], we determined the presence of HPV infection and HPV types in the nasal polyps of patients affected by SNP and the possible effect on HLA-G expression.

## 2. Materials and Methods 

### 2.1. Population

A total of 20 subjects who met the diagnostic criteria of nasal polyps were recruited from the Operative Unit of Otolaryngology, St. Anna Hospital, Ferrara, between the years 2010 and 2013. Among these patients, 15 were male and 5 were female. The median age of these patients was 52.8 years (range: 37–84 years, SD 15.2). All of the 20 patients underwent surgery for the removal of nasal polyps at least once, and in 11 cases such surgery was performed twice. Ten patients presented with allergic diseases (SNP-WAD) while 10 patients did not present with any allergic diseases (SNP-WoAD). All the patients were followed for almost two years from the first surgery, in order to identify the occurring relapses. All the polyps resulted to be of edematous type.

In addition, 10 healthy individuals surgically treated for rhinoplasty (6 males and 4 females) with a median age of 47.5 years (range: 34–79 years, SD 10.2) were included.

Nasal polyps from patients or middle turbinate mucosa from healthy subjects were removed and used for experiments.

All subjects agreed to participate in this study by providing written informed consent (University of Ferrara Ethical Committee Protocol N° 140194).

### 2.2. Nasal Biopsies

Nasal biopsies were collected from inferior turbinates in controls or from nasal polyps in SNP patients. The nasal biopsies were freshly processed to isolate nasal epithelial cells as previously described [28]. Polyps were washed in DMEM and incubated with 0.1% collagenase in DMEM-F12 supplemented with 100 IU/mL of penicillin/streptomycin at 4°C overnight. After incubation, the nasal epithelial cells were isolated by gentle agitation. The residual polyp tissue was discarded. After centrifugation at 1200 rev/min for 5 min, the supernatant was removed and 1 mL of pure FCS was added to the cell pellet to neutralize the enzyme. After further centrifugation, the cells were suspended in DMEM-F12 supplemented with antibiotics and 10% FCS. The dissociated cells were used to separate epithelial cells.

### 2.3. Epithelial Cell Purification

Epithelial cells were isolated from polyps dissociated cells by anti-EpCAM (Ber-EP4) coated immune-magnetic beads (Dynal CELLection Epithelial Enrich, Dynal AS, Oslo, Norway).

Epithelial and residual cells were grown for 5 days in DMEM-F12 medium added with penicillin/streptomycin, Hepes buffer, L-glutamine, and 10% FBS.

### 2.4. DNA Extraction and HPV-PCR

Genomic DNA was extracted from epithelial and residual cells using DNA sorb-B extraction kit (Sacace Biotechnologies, Como, Italy), according to the manufacturer's instructions. The presence of HR-HPV (HPV-16, -18, -31, -33, -35, -39, -45, -52, -53, -56, -58, -59, -66, and -70) and LR-HPV (HPV-6 and HPV-11) was investigated by PCR using the HPV high-risk screen amplification kit and the HPV6/11 screen amplification kit (Sacace Biotechnologies).

### 2.5. Flow Cytometry

HLA-G and IL-10R expression on polyp cells was monitored by immunofluorescence assay with anti-HLA-G (87G-Alexa Fluor 488, Exbio, Praha, CZ) and anti-IL-10R (anti-IL-10RPE, Chemicon, Millipore, MA, USA) moAbs. Anti-isotype controls (Exbio, Praha, Czech Republic) were performed. Cells viability was assessed by propidium iodide staining. Cells were analyzed by a flow cytofluorimetric approach with FACSCount flow cytometer (Becton Dickinson, San Jose, CA, USA) using standard settings and CellQuest software (Becton Dickinson, San Jose, CA, USA) for data analysis.

### 2.6. Immunofluorescence

Adherent epithelial cells and adherent residual cells were analyzed by immunofluorescence with anti-Mucin8-FITC or fibroblast marker-FITC (Santa Cruz Biotechnology, TX, USA) and anti-HLA-G 87G-PE (Exbio). All samples were observed under a UV light microscope (Nikon Eclipse TE2000S, Nikon, Italy).

### 2.7. sHLA-G Enzyme-Linked Immunosorbent Assay (ELISA)

sHLA-G levels in epithelial cell culture supernatants were assayed in triplicate as previously reported [29–31] using, as capture antibody, the monoclonal antibody (MoAb) MEM-G9 (Exbio), which recognizes the HLA-G molecule, in *β*2-microglobulin associated form. The intra-assay coefficient of variation (CV) was 1.4% and the interassay CV was 4.0%. The limit of sensitivity was 1.0 ng/mL.

### 2.8. IL-10 ELISA

IL-10 concentrations were determined in triplicate using the commercial Human IL-10 BioSource immunoassay kit (Human IL-10 US, BioSource, Camarillo, CA, USA) with a detection limit of 0.2 pg/mL.

### 2.9. Statistical Analysis

The samples were analyzed with odds ratio, logistic regression, and Student's *t*-test. Significant *P* values were considered <0.05.

## 3. Results

### 3.1. Presence of HPV-11 Infection in Polyp Biopsies from Relapsing SNP-WoAD Patients

We detected the presence of LR-HPV-11 in 50% (5/10) of samples obtained from patients with SNP-WoAD. Interestingly, these 5 patients presented with a relapsing SNP-WoAD. The other 5 SNP-WoAD did not preset LR-HPV infection and were not characterized by a relapsing disease. None of the SNP-WoAD patients presented with HR-HPV infection. We found no LR-HPV and HR-HPV infection neither in biopsies obtained from SNP-WAD patients nor in healthy controls. These results sustain a role for HPV-11 in the development of relapsing SNP-WoAD (OR: 5.5, 95%, CI: 0.8–38.7). The logistic regression analysis sustains the role of HPV-11 infection as a risk factor for the development of relapsing SNP-WoAD, regardless of gender, age, disease course, and number of relapses (*P* = NS).

### 3.2. Presence of HPV-11 Infection in the Epithelial Fraction of Polyps from Relapsing SNP-WoAD Patients

**Figure 1:**
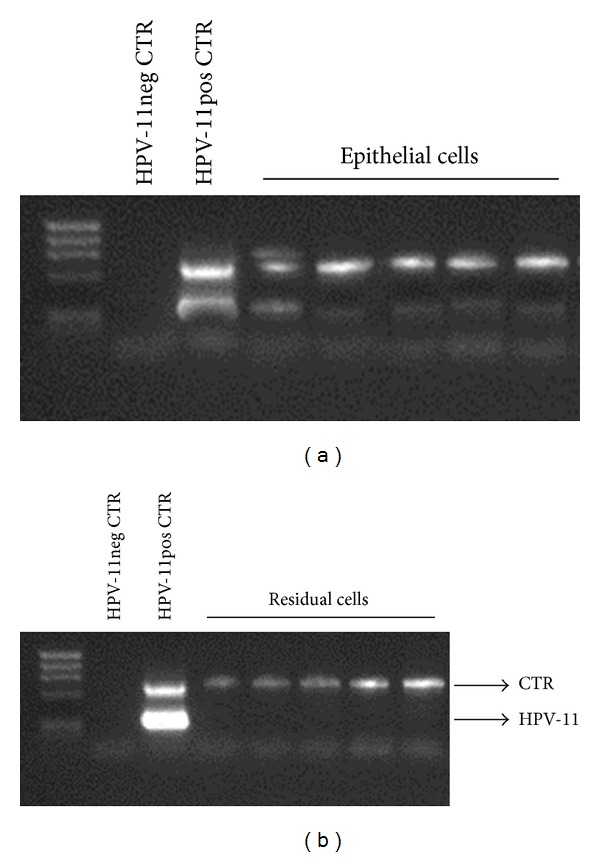
PCR products obtained from HPV-11 DNA analysis by HPV6/11 screen amplification kit (Sacace Biotechnologies) of (a) epithelial cells and (b) residual cells from polyps of the 5 relapsing SNP-WoAD patients. Internal control at 723 bp, HPV-11 at 425 bp. The kit has a sensitivity of 500 copies/mL.

In order to assess the polyp cell fraction infected by HPV-11, we processed the HPV-11 positive biopsies and purified the epithelial cells. The positivity for the presence of HPV-11 was maintained only in the fraction containing epithelial cells (Figure 1(a)). Conversely, the residual fraction was not positive for the presence of HPV-11 DNA (Figure 1(b)).

### 3.3. HLA-G Expression in the Epithelial Fraction of Polyps from Relapsing SNP-WoAD Patients

**Figure 2:**
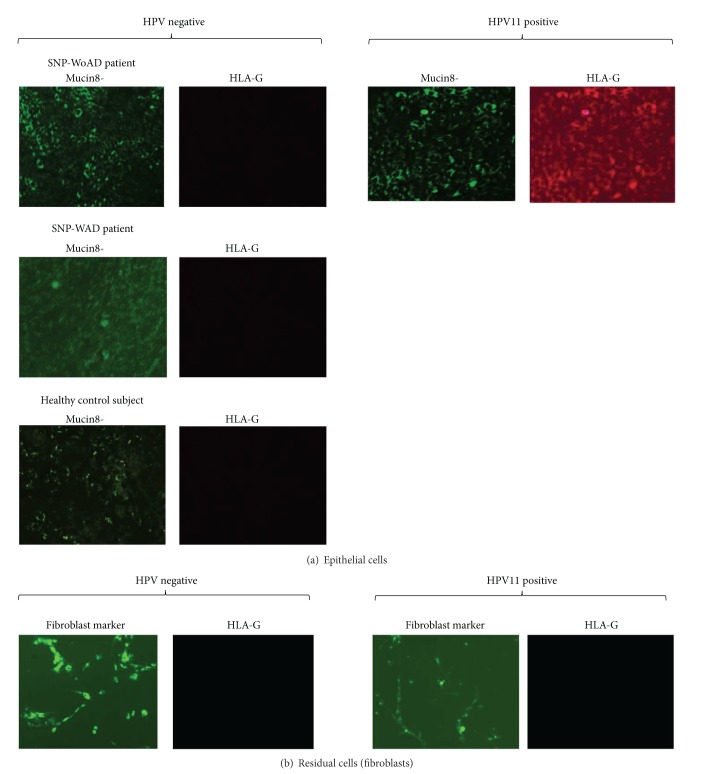
Immunofluorescence analysis of (a) epithelial cells and (b) residual cells (fibroblasts) from representative SNP-WoAD and SNP-WAD patients and controls. The cells were stained with anti-Mucin8-FITC or fibroblast marker-FITC (Santa Cruz Biotechnology) and 87G-PE (Exbio).

Since HPV infection modifies HLA-G expression [10, 11, 25–27], we analyzed the different fraction of cells extracted from nasal polyps for HLA-G expression. Only epithelial cells (Mucin 8^positive^) arising from HPV-11 positive relapsing SNP-WoAD patients showed HLA-G membrane expression (Figure 2(a)). Conversely, the epithelial cells from biopsies of SNP-WoAD patients without HPV infection, of SNP-WAD patients and of healthy controls, presented with no HLA-G expression (Figure 2(a)). In the residual fraction obtained after the purification of epithelial cells, which consisted in fibroblast cells (fibroblast marker^positive^), no HLA-G expression was found in all the three groups of subjects (Figure 2(b)).

### 3.4. sHLA-G and IL-0 Expression in the Epithelial Fraction of Polyps from Relapsing SNP-WoAD Patients

**Figure 3:**
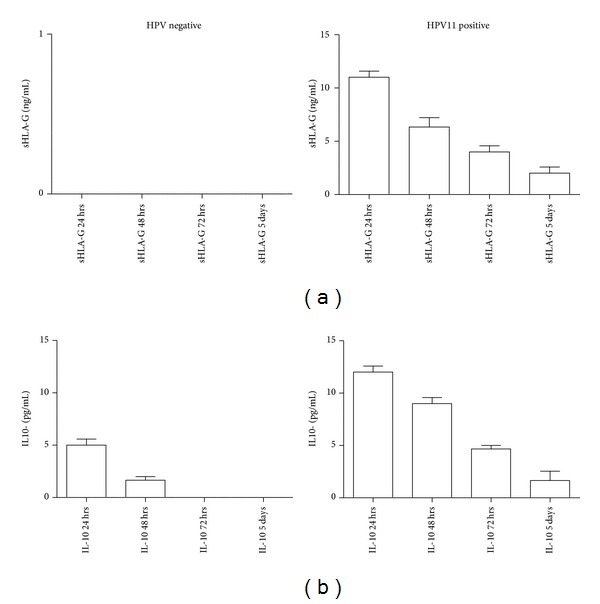
(a) sHLA-G and (b) IL-10 levels in epithelial cell culture supernatants from HPV negative and HPV-11 positive SNP-WoAD patients during a 5-day in vitro culture.

Since HLA-G molecules are present in both membrane and soluble isoforms, we analyzed the epithelial cell culture supernatants from SNP-WoAD for sHLA-G presence. We found the presence of sHLA-G molecules only in HPV-11 positive samples (Figure 3(a)), while no sHLA-G was observed in HPV negative samples (Figure 3(a)) and in all the fibroblasts cultures (data not shown). Interestingly, the secretion of sHLA-G decreased over 5 days of culture. Since IL-10 is known to be an inducer of HLA-G expression and we proposed an implication in SNP [32], we analyzed the levels of IL-10 in epithelial cell culture supernatants. We documented the secretion of IL-10 in both HPV-11 positive and HPV negative epithelial cell cultures (Figure 3(b)). In HPV negative samples, IL-10 decreased after 48 hrs while it lasted for 5 days of culture in HPV-11 positive samples (Figure 3(b)).

**Figure 4:**
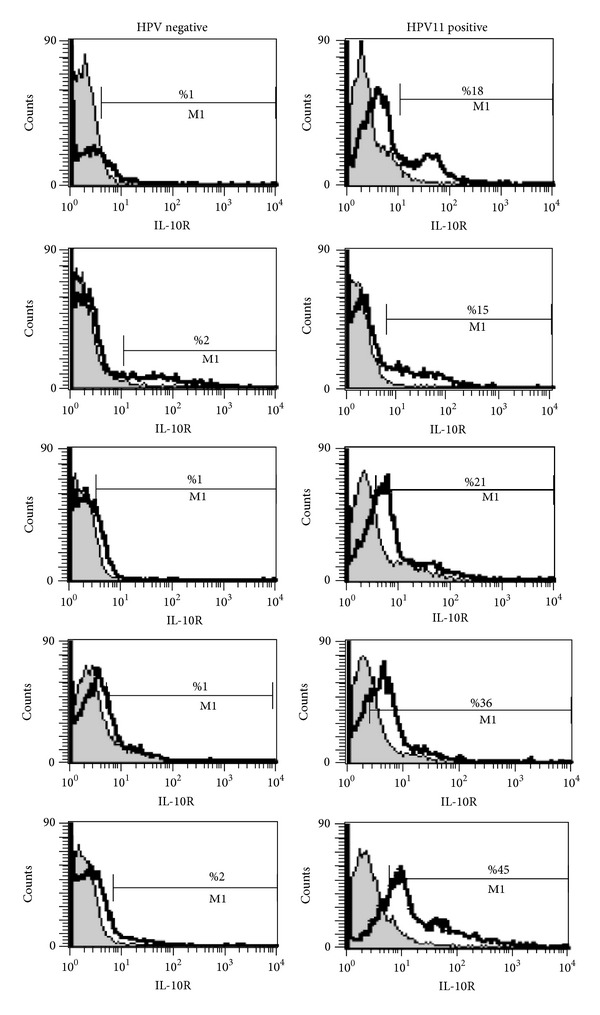
IL-10R expression on epithelial cells from HPV negative and HPV-11 positive SNP-WoAD patients. Grey histogram: anti-isotype controls (Exbio) and white histogram: IL-10R (anti-IL-10RPE) (Chemicon).

Since IL-10 interacts with a specific receptor (IL-10R) at the cell surface, we analyzed its expression on the cells extracted from polyp biopsies. We observed IL-10R on the 27% (median) of epithelial cells from SNP-WoAD patients with HPV-11 infection (Figure 4), while only the 1.4% of epithelial cells from SNP-WoAD patients without HPV infection expressed this receptor (Figure 4) (*P* = 0.0022, Student's *t*-test).

## 4. Discussion

Previous studies in literature have analyzed the role of HPV infection in SNP, demonstrating different correlation ranges [5, 6, 33]. We hypothesize that these differences may be due to the absence of a preselection of patients according to the presence or absence of allergic diseases that could be a confounder for the results observed. In our study, we enrolled the patients on the basis of the characteristics of SNP. Interestingly, the 50% of the SNP-WoAD patients showed positivity for HPV-11 infection and a relapsing disease. On the contrary, HPV infection was absent in SNP-WAD patients and in SNP-WoAD patients without a relapsing disease. These results confirm the importance of the methodological approach in the selection of patients for the study of this pathology and support previous results [33], where HPV-11 was observed as the prevalent infection SNP. Interestingly, HPV infection was restricted to epithelial cells, as it is known to be highly tropic for epithelial cells, while no infection sign was evidenced in fibroblast cells.

The presence of allergic diseases could increase the risk of SNP because of the creation of an inflammatory nasal environment. On the contrary, in the absence of an allergic background, the causes could be found in different environmental factors. In particular, we observed the presence of HPV-11 infection only in relapsing SNP patients, where the persistence of a viral infection could worsen the disease follow-up.

We then evaluated the immunological mechanisms which can be the basis of the virus effect on SNP-WoAD aetiopathogenesis. Interestingly, we found HLA-G expression in HPV-11 positive SNP-WoAD patients, with a secretion of HLA-G molecules that lasted for 5 days of culture. The same behavior was followed by IL-10 that was mainly present in epithelial cell culture supernatants of SNP-WoAD patients. The presence of HPV-11 infection upmodulated also IL-10R on epithelial cells, suggesting a direct role of IL-10 in controlling HLA-G expression. On the contrary, the low IL-10R expression in HPV negative samples could explain the absence of HLA-G expression, even in the presence of IL-10 secretion. Interestingly, we previously reported the absence of sHLA-G production by peripheral blood mononuclear cells (PBMCs) from SNP-WoAD patients after lipopolysaccharide activation and reported IL-10 secretion after PBMCs lipopolysaccharide activation but no induction of HLA-G expression that was restored only after exogenous IL-10 addition [32]. These differences in HLA-G/IL-10 expression pattern between PMBCs and polyp epithelial cells from SNP-WoAD patients support the involvement of HPV-11 infection in local HLA-G and IL-10 induction. In fact, previous results documented the ability of HPV-11 E6 in inducing IL-10 expression [34]. HPV E6 gene is one of the most relevant viral gene products that contribute to the immortalization and transformation of HPV-infected cells [35]. The activation of E6 appears to be critical for in vivo induction of epithelial hyperplasia [36] and thus may lead to the recurrence of nasal polyps. On the basis of our results, we can hypothesize that HPV infection can modify the immune control, possibly via E6, creating a Th2 environment that maintains the chronicization of the infection. We suggest a role for IL-10 and HLA-G molecules, as component of the immune-modulatory cell mechanism that is exploited by the virus to escape the host immune response.

In conclusion, this is the first study elucidating the possible involvement of IL-10/HLA-G feedback loop in maintaining HPV infection in nasal polyps from SNP-WoAD patients. This modification in immune regulation could be at the basis of SNP-WoAD relapsing follow-up. Further studies will be necessary to evaluate the possible role of HPV-11 E6 in inducing IL-10 and consequently HLA-G in this specific pathology. The confirmation of our results could suggest HPV-11 and HLA-G/IL-10 presence as prognostic markers in the follow-up of SNP-WoAD.
